# Experiential Virtual Scenarios With Real-Time Monitoring (Interreality) for the Management of Psychological Stress: A Block Randomized Controlled Trial

**DOI:** 10.2196/jmir.3235

**Published:** 2014-07-08

**Authors:** Andrea Gaggioli, Federica Pallavicini, Luca Morganti, Silvia Serino, Chiara Scaratti, Marilena Briguglio, Giulia Crifaci, Noemi Vetrano, Annunziata Giulintano, Giuseppe Bernava, Gennaro Tartarisco, Giovanni Pioggia, Simona Raspelli, Pietro Cipresso, Cinzia Vigna, Alessandra Grassi, Margherita Baruffi, Brenda Wiederhold, Giuseppe Riva

**Affiliations:** ^1^Applied Technology for Neuro-Psychology LabIstituto Auxologico ItalianoMilanItaly; ^2^Department of PsychologyCatholic University of Sacred HeartMilanItaly; ^3^Institute of Clinical Physiology (IFC)National Research Council (CNR)MessinaItaly; ^4^Virtual Reality Medical InstituteBrusselsBelgium

**Keywords:** psychological stress, Interreality, virtual reality, biosensors, heart rate, heart rate variability, biofeedback training, relaxation training, physiological monitoring, smartphones

## Abstract

**Background:**

The recent convergence between technology and medicine is offering innovative methods and tools for behavioral health care. Among these, an emerging approach is the use of virtual reality (VR) within exposure-based protocols for anxiety disorders, and in particular posttraumatic stress disorder. However, no systematically tested VR protocols are available for the management of psychological stress.

**Objective:**

Our goal was to evaluate the efficacy of a new technological paradigm, Interreality, for the management and prevention of psychological stress. The main feature of Interreality is a twofold link between the virtual and the real world achieved through experiential virtual scenarios (fully controlled by the therapist, used to learn coping skills and improve self-efficacy) with real-time monitoring and support (identifying critical situations and assessing clinical change) using advanced technologies (virtual worlds, wearable biosensors, and smartphones).

**Methods:**

The study was designed as a block randomized controlled trial involving 121 participants recruited from two different worker populations—teachers and nurses—that are highly exposed to psychological stress. Participants were a sample of teachers recruited in Milan (Block 1: n=61) and a sample of nurses recruited in Messina, Italy (Block 2: n=60). Participants within each block were randomly assigned to the (1) Experimental Group (EG): n=40; B1=20, B2=20, which received a 5-week treatment based on the Interreality paradigm; (2) Control Group (CG): n=42; B1=22, B2=20, which received a 5-week traditional stress management training based on cognitive behavioral therapy (CBT); and (3) the Wait-List group (WL): n=39, B1=19, B2=20, which was reassessed and compared with the two other groups 5 weeks after the initial evaluation.

**Results:**

Although both treatments were able to significantly reduce perceived stress better than WL, only EG participants reported a significant reduction (EG=12% vs CG=0.5%) in chronic “trait” anxiety. A similar pattern was found for coping skills: both treatments were able to significantly increase most coping skills, but only EG participants reported a significant increase (EG=14% vs CG=0.3%) in the Emotional Support skill.

**Conclusions:**

Our findings provide initial evidence that the Interreality protocol yields better outcomes than the traditionally accepted gold standard for psychological stress treatment: CBT. Consequently, these findings constitute a sound foundation and rationale for the importance of continuing future research in technology-enhanced protocols for psychological stress management.

**Trial Registration:**

ClinicalTrials.gov: NCT01683617; http://clinicaltrials.gov/show/NCT01683617 (Archived by WebCite at http://www.webcitation.org/6QnziHv3h).

##  Introduction

The emerging convergence of technology and health care [1] is offering new methods and tools for mental health treatments [2-6]. An emerging trend is the use of virtual reality (VR) within the exposure-based protocols for anxiety disorders and posttraumatic stress disorders (PTSD) [7-11]. PTSD is more difficult to treat than other anxiety disorders. On one hand, in vivo exposure-based therapy is usually not possible. On the other, imaginal exposure requires that the patient recounts their traumatic experience in the present tense to the therapist—a behavior that patients try to avoid [12]. VR therapy allows exposure treatment even with patients who fail to improve with traditional imaginal exposure therapy [13-16]. Since the initial work by Barbara Rothbaum and her team [17,18], additional case studies [19-23] and clinical trials [22,24,25], including a randomized controlled clinical trial [26], have shown the efficacy of VR therapy in the treatment of PTSD.

One factor that may increase the likelihood of developing the symptomatology of PTSD is work-related stress. Previous studies have included stressful life events in the list of risk factors for PTSD, suggesting that experiencing chronic psychological stress may increase vulnerability to this anxiety disorder [27,28]. Indeed, chronic psychological stress may induce plasticity within the amygdala, which in turn may increase the risk of developing chronic anxiety states [29]. This abnormal change in the limbic neural circuitry may provoke a pathological anxiety response, leading to syndromes such as PTSD. Other studies have focused on the contribution of work-related stressors to PTSD [30,31]. For example, Laposa et al [30] found that interpersonal conflicts, inadequate support from superiors, and changing jobs were significantly associated with PTSD symptoms. Different training has been developed for managing psychological stress at work, both individually and organizationally focused [32,33]. Specifically, a recent review showed that individual interventions, like cognitive behavioral therapy (CBT), can improve individuals’ mental health, while physical activity as an organizational intervention is more effective in reducing absenteeism [32]. On the basis of this evidence, we decided to focus our intervention on the individual.

However, until now, no systematically tested VR protocols have been available for the management of psychological stress. A preliminary attempt, developed by the US Army, was tested for the training of military medical professionals who are expected to take care of the wounded in very austere situations. This protocol included a technology-assisted relaxation training merging VR exposure sessions with relaxing videos with embedded English narratives guiding progressive muscle relaxation and controlled breathing [34].

According to Cohen et al [35], psychological stress occurs when people perceive that environmental demands tax or exceed their adaptive capacity. In this view, stressful experiences are conceptualized as person-environment transactions, whose results are dependent on the impact of the external stimulus. This is mediated by:

- The person’s appraisal of the stimulus: when faced with a stimulus, a person evaluates the potential threat (primary appraisal). Primary appraisal is a person’s judgment about the significance of a stimulus as stressful, positive, controllable, challenging, or irrelevant.
- The personal, social, and cultural resources available: facing a significant stimulus, a second appraisal follows, which is an assessment of the individual’s coping resources and options. Secondary appraisals address what one can do about the situation.
- The efficacy of the coping efforts: if required by the appraisal process, the individual starts a problem management phase aimed at regulation of the external stimulus.

Stress Management Therapy can help counter effects of psychological stress. Usually various techniques are used including relaxation, interaction, biofeedback, and CBT methods. According to the Cochrane Database of Systematic Reviews [36-38], the best validated approach covering both stress management and stress treatment is the CBT approach. Typically, a CBT protocol (10-15 sessions) includes both problem-focused (eg, resource optimization and better planning) and emotion-focused (eg, relaxation training, use of emotional support) coping strategies. Initially, it includes in-session didactic materials and experiential exercises and out-of-session assignments (practicing relaxation exercises and monitoring stress responses).

The clinical intervention primarily focuses on (1) learning how to cope better with daily stressors (psychological stress) or traumatic events (PTSD), and (2) optimizing one’s use of personal skills and social resources.

The trouble with managing psychological stress is that it is very personal. So the focus for assessment, prediction, and treatment has to be the situated experience of the individual. This result is difficult to achieve using the available VR protocols for PTSD. From a clinical viewpoint, in these protocols VR provides a “closed” experience, separated from the emotions and behaviors experienced by the patient in the real world. In other words, VR exposure tries to change cognitive content per se, rather than changing the context in which cognitions are experienced [39]. The behavior of the patient in VR has no direct effects on the real-life experience. The emotions and problems experienced by the patient in the real world are not directly addressed in the VR exposure. Moreover, it focuses on patients’ thoughts and behaviors but does not address social support and coping skills.

To overcome these issues, Riva et al [40-42] suggested the use of the “Interreality” paradigm (IR) that integrates assessment and treatment within a hybrid environment, bridging the physical and virtual world. The basic idea of the IR intervention is to bridge virtual experiences (fully controlled by the therapist, used to learn healthy behaviors and coping skills) with real experiences (the therapist can identify critical situations and assess clinical change). In the IR strategy, behavior in the physical world influences the experience in the virtual one, and behavior in the virtual world influences the experience in the real one. The current CBT approach can be described as imagining evokes emotions and the meaning of the associated feelings can be changed through reflection and relaxation. In IR, we introduce an alternative strategy, in which controlled experiences evoke emotions that result in meaningful new feelings, which can be reflected on and eventually changed through reflection and relaxation. This is achieved by using technology (virtual worlds, advanced sensors, and smartphones) to create a closed-loop approach for assessment and support.

The assessment is conducted continuously in the virtual and real worlds through the tracking of individuals’ behavioral and emotional status over time, in the context of realistic task challenges. At the same time, the information is constantly used to improve both the appraisal and the coping skills of the patient through a conditioned association between effective performance state and task execution behaviors.

**Figure 1:**
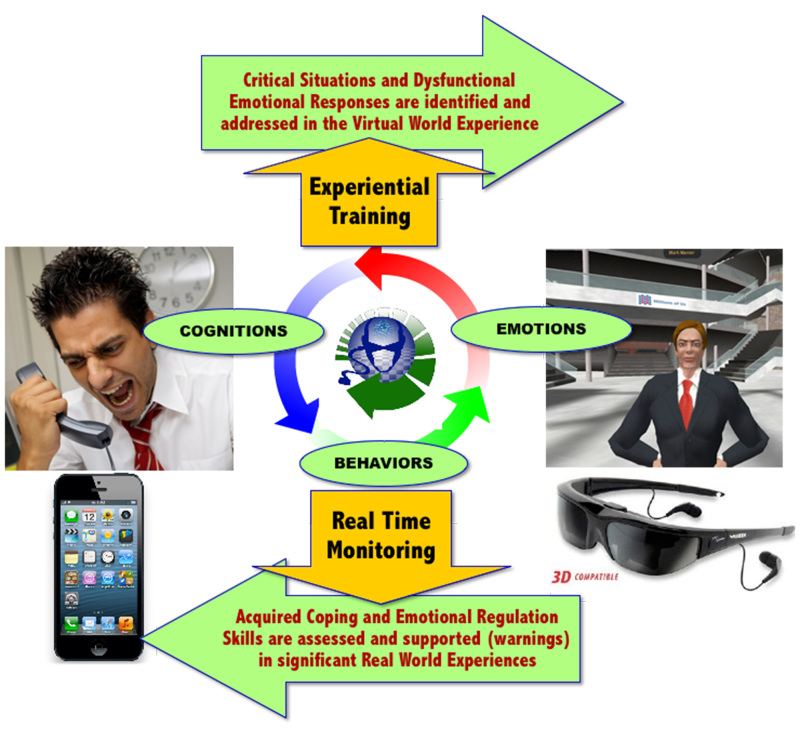
Advantages of Interreality.

**Figure 2:**
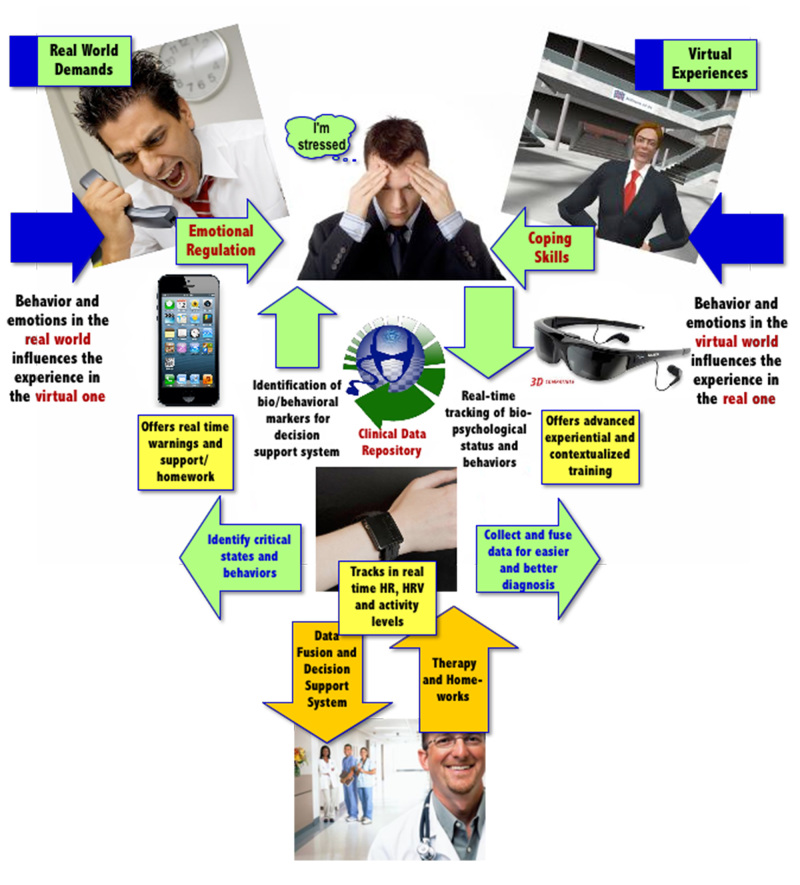
The Interreality paradigm for the management of psychological stress.

These features are integrated into two subsystems: the clinical VR platform (VR inpatient treatment, fully controlled by the therapist) and the mobile platform (mobile-based real world support, available to the patient and connected to the therapist). Combined, these systems are able to provide (1) objective and quantitative assessment of symptoms using biosensors and behavioral analysis: monitoring of the patient behavior and of general and psychological status, early detection of symptoms of critical evolutions, and timely activation of feedback in a closed loop approach, and (2) decision support for treatment planning through data fusion and detection algorithms: the decision support system allows monitoring stress levels of the patient both during VR exposure (in the therapist’s office) and in real-life situations (using the mobile phone), by generating reports that the therapist can access via a Web-based interface. The key features of IR are summarized in Figures 1 and 2.

Simply put, patients are continuously assessed in the virtual and real worlds by tracking their behavioral and emotional status in the context of challenging tasks (customization of the therapy according to the characteristics of the patient), and feedback is continuously provided to improve patients’ skills (improvement of self-efficacy).

The potential clinical advantages of the IR strategy are (1) an integrated and quantitative assessment of the user’s stress level using biosensors: the level of stress is continuously assessed in the virtual and in the real world by recording the participant's behavioral and emotional status for the decision support system, and (2) provision of warnings and motivating feedback to improve self-awareness, compliance, and long-term outcomes: on the basis of the decision support system, participants constantly receive feedback to improve their appraisal and coping skills in an entertaining and motivating manner both in clinical and mobile settings [40-42].

Starting from the above premise, the main goals of this study are (1) to define and develop an Interreality protocol for the management of psychological stress, and (2) to compare, within a controlled study, its efficacy with a similar non-technological protocol based on the CBT approach.

We hypothesize that the Interreality protocol is more effective than both standard CBT and a wait-list condition in (1) reducing the level of chronic “trait” stress, (2) reducing the perceived stress and improving quality of life, and (3) improving the coping skills of the individual.

##  Methods

### Recruitment

**Figure 3:**
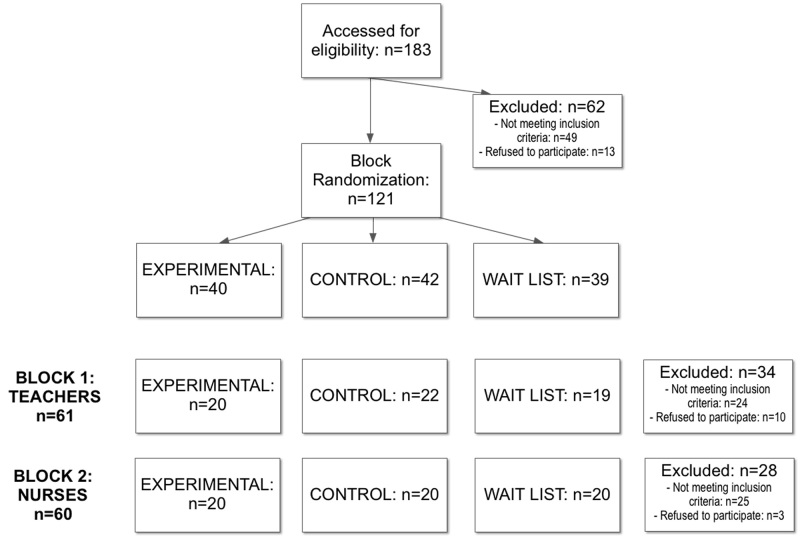
Consort flowchart.

The study is designed as a multicentric randomized block controlled trial involving participants recruited from two different worker populations (teachers and nurses) that are highly exposed to psychological stress (the Consort flowchart is reported in Figure 3 and the electronic CONSORT-EHEALTH questionnaire [43] in Multimedia Appendix 1). Compared to a completely randomized design, this design reduces variability within treatment conditions and potential confounding (the variability within blocks is less than the variability between blocks), producing a better estimate of treatment effects [44].

A sample of high school teachers recruited in Milan (Block 1: n=95) and a sample of pediatric nurses recruited in Messina, Italy (Block 2: n=88) were seen for screening interviews for admission to the study. Samples were recruited between March 2012 and September 2013. Criteria for participation included the following: (1) a high level of perceived stress (≥7) as measured on a 10-item visual-analogue scale, (2) a high level of relevance of stress for personal health (≥7) as measured on a 10-item visual-analogue scale, (3) a low level of self-efficacy related to stress management (≤5) as measured on a 10-item visual-analogue scale, (4) no DSM-IV-TR (Diagnostic and Statistical Manual of Mental Disorders, 4^th^ edition, text revision) Axis I disorders as assessed by Mini-International Neuropsychiatric Interview (MINI) [45,46] during the clinical assessment, (5) aged 25-60 years, (6) no psychotherapy received for their psychological stress as assessed with a clinical interview, (7) no current psychiatric medications as assessed with a clinical interview, (8) no history of neurological diseases, psychosis, alcohol or drug dependence as assessed with a clinical interview, and (9) no migraine, headache, or vestibular abnormalities as assessed with a clinical interview.

Both males and females were included. In order to select participants, we decided to use only subjective indexes of stress and coping for this study, without measuring cortisol. The measures of the concentration of cortisol in blood, saliva, and urine are established methods for momentary assessments of the activity in the hypothalamic-pituitary-adrenocortical axis (HPA). If the cortisol levels become too high or too low for a longer period, a state of hyper- or hypocortisolism is present, and both are associated with stress-related disease. However, two recent studies by Barth [47] and Faresjo [48] suggest that cortisol levels are not very reliable indicators of stress. The first study [47] underlined the relevance of subjective evaluations in producing the negative effects of stress. The author found a reduced risk of coronary heart disease in stressed individuals who neglected the subjective relevance of stress on health. The second study [48] demonstrated the inefficacy of measuring cortisol levels for assessing stress in subjects living in a stressful environment. The study found that living in a stressful economic and social environment produced a down-regulation of the HPA-axis with a suppression of cortisol levels.

In our study, 62 participants either did not fulfill inclusion criteria or refused to participate for other reasons (eg, time constraints). This is an unusual observation. However, it should be noted that participants experienced a high level of psychological stress. Chronic psychological stress alters the psychophysiological processes involved in cognitive appraisals and coping responses. Several types of coping strategy are commonly used to face the different demands associated with stressful events, and their effective use reflects variation in underlying cognitive appraisal. This refusal may therefore be read as their inability to appraise and cope with the stressful events.

All patients meeting the inclusion criteria in each block (B1=61, B2=60) were randomly assigned to the three groups: (1) the Experimental Group (EG): n=40, B1=20, B2=20, (2) the Control Group (CG): n=42, B1=22, B2=20, and (3) the Wait-List group (WL): n=39, B1=19, B2=20. All the participants signed an informed consent form before entering the study. The sample characteristics are shown in Table 1.

**Table 1:** Demographic parameters and baseline characteristics of the sample (mean and standard deviation).

Variables		EG	CG	W-L
Age		46.3 (7.7)	42.9 (10.5)	39.6 (9.7)
Years of education		17.9 (1.4)	17.3 (1.4)	18.3 (1.3)
PSM^a^		91.4 (25.8)	86 (19.5)	92.6 (25.8)
PSS^b^		21.1 (7.9)	18 (5.9)	19.4 (6.6)
STAI-Y2^c^		43.6 (11.2)	42.1 (11.1)	40.2 (9.8)
SWLS^d^		23.4 (6.2)	24.2 (7.2)	26.4 (7.0)
**COPE-NIV** ^e^				
	Use of emotional support	27.8 (8.2)	29.9 (7.0)	29.4 (7.7)
	Positive attitude	27.9 (5.5)	29.1 (5.1)	31.8 (4.5)
	Problem focused	28.6 (6.0)	29.2 (7.4)	31.7 (4.6)
	Religious coping	19.8 (5.0)	21.7 (5.4)	23.8 (5.5)
	Denial	24.1 (5.9)	21.8 (4.7)	22.8 (3.8)

^a^Psychological Stress Measure.

^b^Perceived Stress Scale.

^c^State-Trait Anxiety Inventory Form Y-2.

^d^Satisfaction With Life Scale.

^e^Coping Orientation to the Problems Experienced—New Italian Version.

Baseline comparisons among the three groups showed only a difference in the age of the groups. The age was significantly higher in the EG and to a lesser extent in the CG than in the WL group. Of the EG participants, 57.5% were women (23/40) and 42.5% (17/40) were men, while the CG and WL included, respectively: 71.4% women (30/42) and 28.6% men (12/42), and 51.3% (20/39) women and 48.7% men (19/39).

We also assessed participants’ computer literacy at the start of the trial through a self-assessment scale with three values: “low”, “medium”, and “high”. Results showed overall a medium level of perceived individuals’ technological abilities, and no significant differences were found between groups.

### Ethics

The study was approved by ethical review board of Istituto Auxologico Italiano in Milan, Italy, and by the ethical review board of Azienda Ospedaliera Universitaria Policlinico “G. Martino” in Messina. The study was conducted according to the 1964 Declaration of Helsinki. All the participants signed an informed consent form that explained the goal of the treatment, its duration, the involvement of the patients, and for EG individuals, the possible side effects related to the extended use of immersive virtual environments (ie, ocular problems, disorientation and balance disturbances, and nausea).

### Treatment Protocols

The treatment protocols were based on 5 weeks of stress management training (2 sessions per week) following the “stress management program” by Kaluza [49] and on the “stress inoculation training” by Meichenbaum [50].

Stress Management Training (SMT) is a short, focused, and individualized intervention to improve individual coping with stress at workplace [49]. A meta-analysis of 36 studies showed the efficacy of SMT in reducing negative mood states (ie, anxiety, depression) [51]. The Stress Inoculation Training (SIT) [50,52] is a validated short, semistructured, and active approach to manage psychological stress (for a review, see [53]). The effectiveness of SIT has been evaluated in different contexts. In the clinical setting, SIT has proven an effective means of helping patients face particularly strenuous conditions [54-56]. In the occupational environment, SIT has been successfully applied to support employees in managing stressful situations [57,58] and to help athletes manage anxiety and improve performance [11,59].

The treatment block for teachers (Block 1: n=61) was offered by therapists from Istituto Auxologico Italiano both in the Istituto Scientifico Ospedale San Luca, Milano, Italy and in the schools where teachers worked. The treatment block for nurses (Block 2: n=60) was offered by therapists from the Azienda Ospedaliera Universitaria Policlinico “G. Martino” at the Pervasive Healthcare Center, a clinical research center of the Institute of Clinical Physiology.

The IR and CBT treatments were administered by clinical psychologists with extensive experience in stress management techniques. Detailed manuals were prepared to facilitate adherence with the treatment protocols. Clinical reports were checked for compliance at monthly supervision meetings. A high level of protocol adherence was reported by therapists. The difference between the treatment protocols offered by each block is detailed below (see also [41]).

### Experimental Group

#### Overview

**Figure 4:**
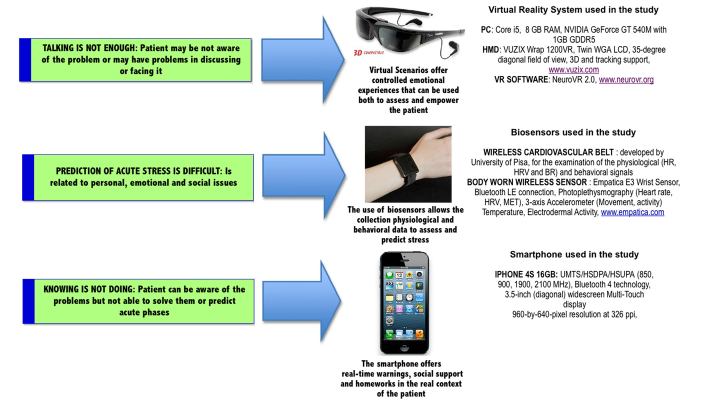
Technologies used by the experimental group.

The experimental group used an Interreality protocol based on the following technologies (see Figure 4 for details).

#### 3D Virtual Scenarios (in the Therapist’s Office)

##### Immersive Role-Playing Scenarios Where the Individual Interacts With Potentially Stressful Experiences

**Figure 5:**
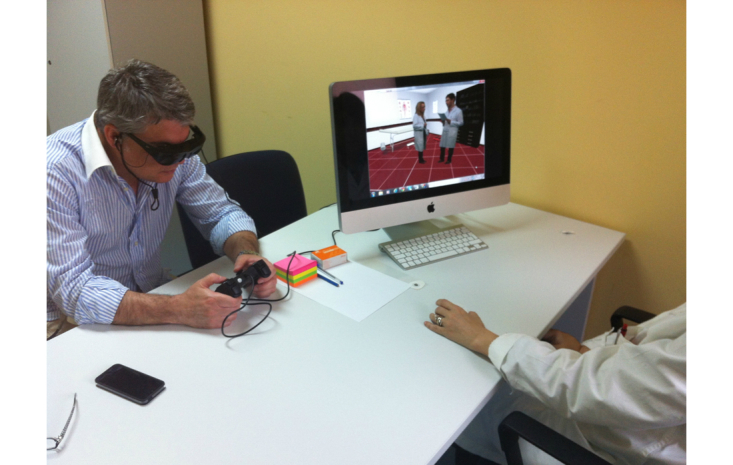
A Virtual Reality treatment session of the Interreality trial.

According to a literature review and the results of a qualitative analysis, different virtual stressful scenarios were generated for both teachers and nurses (see Table 2). The stressful scenarios were played by real actors, video-recorded, and included in the virtual environments (using the free virtual reality platform NeuroVR 2 [60-62]) after post-production (for a demonstration of the procedure, see the video in Multimedia Appendix 2 and Figure 5).

##### Immersive Natural Scenarios Used to Learn Specific Relaxation Techniques

In recent years, VR has been used in different clinical protocols to facilitate relaxation processes in stressed or anxious subjects [63-65] by visually presenting relaxing scenes [34,66]. The IR relaxation environments were created on the basis of similar virtual relaxing environments validated in previous studies [34,64,67-69]. They included a beach, a lake with a waterfall, a campfire in a mountain resort, and a desert oasis.

**Table 2:** The different virtual stressful scenarios.

Virtual scenarios for teachers	Virtual scenarios for nurses
Workload	Managing the patients’ relatives
Class management	Managing patients’ complaints
Coping with parent’s criticism	Managing a medical emergency situation
Relationship with boss	Relationship with colleagues
Coping with parents’ handling efforts	Managing relatives’/caregivers anxiety
Relationship with co-workers	Distribution of work tasks
Conflict management	Patient-doctor communication
	Managing patient’s anxiety
	Unsuccessful collaboration/communication with colleagues
	Discussions among medical doctors

#### Biosensors (Personal Biomonitoring System)

##### Overview

All elements of technology, including smartphone and biosensors, were loaned to the EG participants free of charge. Moreover, all the required data connection fees for real-time stress monitoring were paid for by the trial. All the participants received 1-week group technology training plus personalized support sessions if needed.

##### Biofeedback (in the Therapist’s Office)

Typically, 3D virtual worlds are a closed experience and do not reflect in any way the real activity and status of the users. In IR, physiological sensors (heart rate and heart rate variability) are used to track the emotional/health status of the individual and to influence their experience in the virtual world. To improve the efficacy of the relaxing environment, some features of the experience (eg, the size of the fire or the waterfall flow rate) were driven by the emotional status of the patient as measured by biosensors (heart rate or heart rate variability).

##### Physiological Data Recording (Outside the Therapist’s Office)

To assess the level of contextual stress, each individual was provided a body-worn wireless sensor (EMPATICA E3 wrist sensor) that was able to record and transmit psychophysiological (heart rate and heart rate variation) and activity data in real time.

#### Mobile Phone (Outside the Therapist’s Office)

##### Stress Tracking

The data received from the wireless sensors were assessed in real time by a decision support system (the description of the system that was used can be found in [70,71]). This system provides the user with a graphical representation of the current stress level experienced and allows them to check the history of stress levels variations experienced at different timescales (eg, day, week, month).

##### Contextualized Homework

According to the performance achieved in the therapist’s office and level of stress assessed by the decision support system, the individual was able to experience on the smartphone different guided relaxation and biofeedback virtual experiences (non-immersive) similar to the ones experienced in the therapist’s office.

#### Schema

##### Assessment Session (Session 1)

The session started with a discussion with the clinician about the assessment week. Then, after a brief introduction to the specific content of this session, the psychometric questionnaires were administered for the first time (see below), and the physiological baseline of the participant was recorded for 3 minutes. To measure the psychological variations occurring during the different stressful virtual scenarios, subjects completed the Visual Analogue Scale for Anxiety (VAS-A) and the State-Trait Anxiety Inventory Form Y-1 (STAI-Y1) at the baseline and after each scenario. During stressful exposition, participant physiological parameters were also recorded. Besides the stressful scenarios (see Table 2), each participant was assessed in a neutral virtual environment and in one where they completed a cognitive task in front of a virtual audience. This allowed the therapist to identify the situations inducing the highest level of stress. At the end of this session, the clinician explained to the participant how to use the smartphone and the body-worn wireless sensor.

##### Training Session (Sessions 2-9)

The following sessions were dedicated to teaching participants how to cope with stress, through cognitive restructuring techniques and relaxation exercises. Each session lasted about 1 hour and was divided into four parts: homework checking, exposure to a stressful VR environment, relaxation exercises (with or without biofeedback), and a homework assignment. The clinician decided with the participant the specific stress scenarios to work on in the exposure (cognitive restructuring) during at least two consecutive sessions. Cognitive restructuring was used to help patients identify and challenge their erroneous beliefs and interpretations. Specifically, patients were taught to look at their negative beliefs, look for possible alternative explanations and ways of thinking, and evaluate the pros and cons of maintaining them. Relaxation was induced through the immersion in a natural scenario selected by the subject, where they could move and interact. The experience also integrated different pre-recorded audio narratives describing the specific setting and guiding the execution of different progressive muscle relaxation/deep breathing exercises. The scenario was experienced with or without VR biofeedback, in alternate ways during sessions. At the end of the sessions, the clinician explained to the participant how to use the smartphone and the body-worn wireless sensor to check the level of contextual stress and do the contextualized homework.

##### Follow-up Session (Session 10)

To verify the efficacy of the training, during the final session participants were reassessed through the administration of psychometric questionnaires. Moreover, participants were re-exposed to the different stressful virtual scenarios, following the same procedure of the assessment session (Session 1). At the end of this assessment, the clinician discussed with the participant the protocol and its perceived efficacy/usefulness.

### Control Group

The control group used a protocol based on traditional cognitive behavioral techniques, following the same structure (10 one-hour sessions in 5 weeks) and the same assessment points of the EG, but without the use of any technological tool. Exposure therapy designed for the control group involved imaginal exposure to the same stressful situations reproduced in virtual reality (see Table 2). Patients were instructed to close their eyes and experience the stressful situation by imagining that it was currently happening. An audio recording provided a detailed description of the context, the participants, the physical sensations, and emotional reactions.

Guided imagery was also used to teach relaxation exercises. Like in the EG, the imagery experience was supported by the same pre-recorded audio narratives describing the specific scenario and guiding the execution of different progressive muscle relaxation relaxation/deep breathing exercises.

CG participants did not use the mobile phone for stress assessment, but a traditional diary in which they recorded every relevant stressful event. Moreover, participants’ homework was a self-help book about stress management. Topics included identifying and fully understanding what stress is, how stress affects our performance, the importance of becoming aware of stress, and simple strategies to make desired changes to reduce stress. See Multimedia Appendix 3 for the EG and CG study protocols.

### Wait-List Group

The wait-list group did not receive treatment. They completed all facets of the pre-test and post-test (after 5 weeks), similar to those individuals included in the other groups.

### Assessment

#### Questionnaires

To assess the effects of the stress management protocols, several questionnaires were used at different points of time.

#### Clinical Assessment

To exclude participants suffering from DSM-IV-TR Axis I disorders, the recruited sample was assessed before the start of the training using the MINI semistructured interview [45,46]. The MINI is a short diagnostic, structured interview that enables researchers to make diagnoses of psychiatric disorders according to DSM-IV or the International Statistical Classification of Diseases and Related Health Problems, 10^th^ revision (ICD-10). The administration time of the interview is approximately 15 minutes and was designed for epidemiological studies and multicentered clinical trials.

#### Primary Outcome Measures

##### Overview

The following questionnaires were administered offline under a therapist’s supervision to each participant at pre-treatment, and upon completion of the trial (after 5 weeks).

##### State-Trait Anxiety Inventory Form

State-Trait Anxiety Inventory form (STAI) [72,73] consists of two scales containing 20 items each that measure anxiety in adults. The STAI clearly differentiates between the temporary condition of “state anxiety” (STAI Form Y-1, also known as STAI-Y1) and the more general and long-standing quality of “trait anxiety” (STAI Form Y-2, also known as STAI-Y2). For the initial and the final assessment in the trial, we used the STAI Y2, that is, the trait version of the STAI, which measures characteristic tendencies to be anxious.

##### Coping Orientation to the Problems Experienced—New Italian Version

Coping Orientation to the Problems Experienced (COPE) Inventory was originally developed to assess a broad range of coping responses [74]. COPE-NIV represents a useful and validated tool to assess different coping dimensions in an Italian context [75]. It consist of five scales, for a total of 60 items altogether: use of emotional support, denial, positive attitude, problem focused, and religious coping. This inventory can be used to assess trait coping (the usual way people cope with stress in everyday life) and state coping (the particular way people cope with a specific stressful situation).

##### Perceived Stress Scale

The Perceived Stress Scale (PSS) [76] is a 10-item self-reported measure designed to deal with the degree to which situations in an individual’s life are appraised as stressful. It was originally developed as a 14-item scale that assessed the perception of stressful experiences over the previous month using a 5-point Likert scale. Later, the authors reported that the 10-item version (PSS-10) showed stronger psychometric characteristics in comparison to the 14-item scale [77].

##### Psychological Stress Measure

The Psychological Stress Measure (PSM) [78,79] consists of 49 items based on the various individual perceptions of the cognitive, physiological, and behavioral state of subjects. PSM provides a global score of stress and some partial subscores. Patients are asked to answer on the basis of how they have been feeling in the last 4-5 days. The global score of the PSM is compared with ground truth scores, which give threshold cut-offs on the basis of the gender (103 for male and 110 for female subjects).

##### Satisfaction With Life Scale

The Satisfaction with Life Scale (SWLS) [80,81] is a measure of life satisfaction (subjective well-being). The 5-item questionnaire is designed to measure global cognitive judgments of satisfaction with one’s own life. Qualitative feedback was also obtained using a semistructured questionnaire at pre-treatment, and upon completion of the trial.

#### Secondary Outcome Measures

##### Overview

Individuals were also assessed at the beginning and at the end of each of the 8 training sessions using the following questionnaires.

##### State-Trait Anxiety Inventory Form Y-1

STAI Y1 [72,73] addresses state anxiety, which could be defined as a temporary emotional condition characterized by apprehension, tension, and fear about a particular situation or activity. This inventory consists a of a 20-item scale, like the STAI Y2.

##### Visual Analogue Scale for Anxiety

Visual Analogue Scale for Anxiety (VAS-A) is an instrument that measures anxiety across a continuum. It is a horizontal line, 100 millimeters in length, anchored by word descriptors at each end (No anxiety; Very severe anxiety). Individuals mark on the line the point that they feel represents their perception of their own current state. The VAS-A score is determined by measuring in millimeters from the left end of the line to the point that the person marks.

#### Power Analysis

A power analysis was conducted to determine the appropriate number of participants needed for the current study. With an established alpha level of .05, 0.80 power, and a preliminary research based effect size of 0.272 (n=15, treatment vs non-treatment using the STAI Y2 score), a sample size of 30 participants for the group was enough to test the hypothesis of significant differences between groups. Using the trial sample size of 40 participants for groups, we achieved a power of more than 0.9.

#### Statistical Analysis

Our primary end points are the change of the level of chronic “trait” stress, perceived stress, and coping skills from baseline (pre) to the end of intervention (post). Secondary outcomes included the change in situational “state” stress scores from start (pre) to the end of intervention (post) in each treatment session.

For primary analyses, stress and coping scores were assessed by analysis of covariance (ANCOVA), with post-treatment scores as the baseline variable, while the pre-treatment scores served as covariates. This approach allowed us to *“adjust”* posttest scores for the variability on the pretest ones produced by the block design [82]. Several assumptions were met to use these techniques: (1) the use of interval or ratio data, (2) equally and normally distributed deviations in scores, (3) existence of a linear relationship between the dependent variable and the covariate (pretest scores), and (4) random assignment to groups.

A two-sided *P* value of .05 or less was considered to be statistically significant. For secondary analyses, the experimental and control groups were simultaneously taken into the analysis of variance model for repeated measures (T=8). Differential effects of the treatments were determined using post-hoc analyses. In particular, to reduce the risk of type I errors, we used the Bonferroni post-hoc procedure with an adjusted Experiment-wise Error Rate (EER): 0.05 for each variable in a three-group analysis and 0.025 for each variable in a four-group analysis [83]. Prior to analysis, the distributions for the outcome variables were examined. We detected univariate outliers using boxplots.

##  Results

### Primary Outcome Variables

Outcome data were available for all the participants at the end of treatment (see Table 2). The one-way ANCOVA on the pre-post-treatment scores showed a significant group effect (*F*_2,107_=4.42; *P*<.014; effect size=0.74) on the primary outcome variable of chronic “trait” anxiety (STAI Y2). While no significant differences were found in either the WL group or the CG between pre- and post-measurements, the EG was able to obtain a significant decrease in anxiety (1.2%). These data were confirmed by post-hoc analyses: they revealed significant differences between the EG and the CG (*P*<.05), and between the EG and the WL (*P*<.01).

We then used ANCOVA to analyze the pre-post changes in the level of coping skills. On one side, no significant differences were found in WL between the pre- and post-measurements. On the other, the two treatment groups were able to obtain significant coping skill improvement in four subscales (see Table 2) with a significant greater improvement for the EG (*P*<.05) in the Emotional Support skill. The analysis on the other stress scales (PSS, PSM) revealed a significant reduction in both treatments groups (see Table 3).

Post-hoc analyses did not reveal significant differences among the treatments, although the percentage of decrease in stress scales for the EG was higher (PSS: EG=21%, CG=16%; PSM: EG=13%, CG=5%). No differences were found in the WL group. Finally, we did not find difference between the three groups in the pre-post quality of life (SWLS) scores.

We also compared the two blocks (teachers and nurses) to check for differences. In general, nurses obtained slightly better values than teachers in most of the outcome variables for both treatments. We also found limited but significant differences in the STAI Y2 reduction (Teachers: -0.44+/-8.9; Nurses: -3.2+/-5.3; *P*=.44, effect size=0.04), in the COPE-NIV Emotional Support skill increase (Teachers: -0.3+/-6.3; Nurses: 2.3+/-5.4; *P*=.20, effect size=0.05) and in the SWLS quality of life increase (Teachers: -0.6+/-5.58; Nurses: 1.8+/-3.3; *P*=.005, effect size=0.07).

**Table 3:** ANCOVA results.

Variables			Group			ANCOVA			
			Experimental	Control	Waiting List	*F*	df	*P*	*η* ^*2*^
**STAI Y2**									
		Time 1	43.6 (11.2)	42.1 (11.1)	39.8 (8.71)	4.42	2, 107	.014	.74
		Time 2	38.2 (8.09)	41.8 (10.8)	40.2 (8.82)				
**COPE-NIV**									
	Use of emotional support								
		Time 1	27.8 (8.23)	29.9 (7.04)	29.4 (7.17)	17.2	2, 111	<.001	.237
		Time 2	31.7 (7.62)	29.5 (6.82)	28.8 (7.59)				
	Positive attitude								
		Time 1	27.9 (5.54)	29.1 (4.69)	31.7 (4.08)	16.7	2, 109	<.001	.234
		Time 2	31.4 (5.16)	29.4 (5.86)	30.9 (4.01)				
	Problem focused								
		Time 1	28.6 (5.96)	29.2 (7.36)	31.3 (3.78)	6.34	2, 109	.002	.104
		Time 2	31.7 (7.62)	29.5 (6.48)	29.6 (6.02)				
	Religious coping								
		Time 1	19.8 (5.01)	21.7 (5.39)	23.8 (5.63)	2.81	2, 111	.064	.048
		Time 2	21.1 (6.65)	21.3 (5.63)	23.2 (6.1)				
	Denial								
		Time 1	24.1 (5.91)	21.8 (4.69)	23.1 (3.69)	18.6	2, 116	.009	.082
		Time 2	22.3 (5.13)	20.9 (4.28)	24 (5.27)				
**PSS**									
		Time 1	21.1 (7.95)	18 (5.94)	19.5 (6.64)	10.1	2, 111	<.001	.155
		Time 2	16. 6 (4.75)	15 (5.55)	19.8 (7.24)				
**PSM**									
		Time 1	91.4 (25.8)	86.1 (19.5)	92.3 (26.1)	2.2	2, 111	.115	.39
		Time 2	79.2 (18.3)	81.6 (19.8)	92.4 (23.2)				
**SWLS**									
		Time 1	23.4 (6.22)	24.2 (7.18)	26.5 (6.95)	3.43	2, 111	.036	.058
		Time 2	25.5 (6.57)	24.5 (7.53)	23.2 (6.1)				

### Secondary Outcome Variables

To better understand the differences between the EG and CG, we used a repeated measures analysis of variance (ANOVA) (T=8) to analyze the changes in the situational “state” stress scores (STAI Y1 and VAS-A) before and after each treatment.

**Figure 6:**
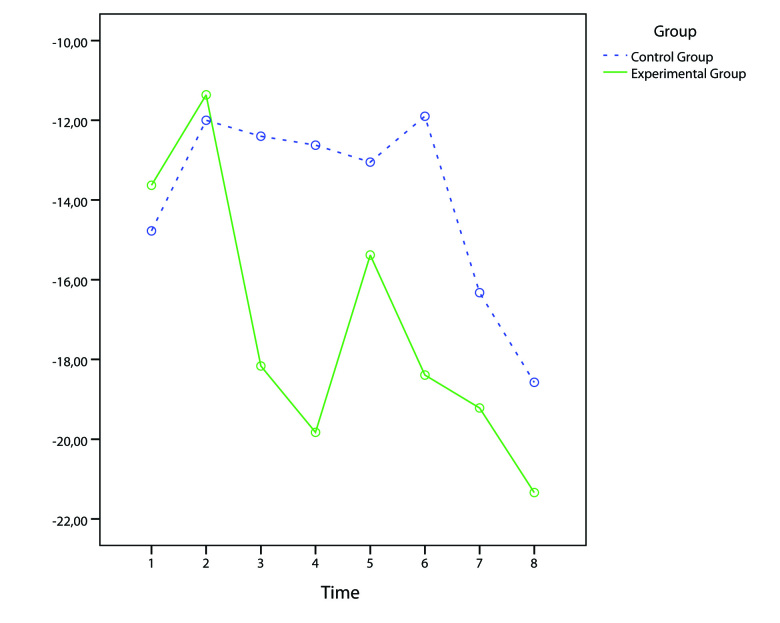
Mean VAS-A reduction (pre-post) in the 8 treatment sessions for both treatment groups.

The analysis revealed significant TIME (*F*_7,511_=3.8; *P*<.01; effect size=0.05) and TIME x GROUP (Quadratic contrast: *F*_1,73_=4.23; *P*<.05; effect size=0.05) effects in the VAS-A scores. First, both groups progressively increased the relaxation level achieved in a session during the protocol. Second, the pattern of this increase is curvilinear (ie, is represented by a curve with one bend) with a difference between the two groups (see Figure 6). EG showed a marked (>15%) early increase in the relaxation level (from Session 3) and a further improvement (>20%) in the next sessions; CG instead showed an initial increase of the relaxation level (>10%) from the first session, with a marked (>15%) increase in the relaxation level only in the last sessions (7-8). The visible peaks experienced by EG in Figure 6 correspond to the introduction of a new stress scenario used by the clinician for VR exposure (see description of the training sessions).

The analysis of STAI scores revealed a similar pattern but with a lack of significant values due to the low statistical power (0.23). We then compared the two blocks (teachers and nurses) to check for differences. Again, nurses obtained higher, but not significant, relaxation values than teachers in all the sessions.

##  Discussion

### Principal Results

Although both treatments (CBT and IR) were able to significantly reduce perceived stress (with a better outcome for IR), only participants who received IR reported a significant reduction (12% vs 0.5%) in chronic “trait” anxiety. This outcome is also better than that (11% reduction) obtained in a previous trial by a 5-week stress management meditation program where self-selected teachers were instructed to use meditation twice daily for 20-minute periods, both at school and at home [84]. In the IR protocol, no compulsory relaxation exercises were required outside the therapist’s office, which is an obvious advantage for both unmotivated individuals and for individuals who are unable to understand the meditation technique or to apply it properly.

A similar pattern was found for coping skills. Both treatments were able to significantly increase most coping skills, but participants who received IR reported a significantly greater increase (14% vs 0.3%) in the Emotional Support skill than CBT. It is interesting to note that meditation/mindfulness stress management programs, unlike CBT ones, do not address coping skills in their protocol. However, different clinical trials showed long-term efficacy of coping skill training for better emotional control [85], quality of life [86,87], and resiliency enhancement [88].

In sum, the obtained data suggested both the clinical efficacy of IR and its enhanced efficacy over CBT and other existing protocols in the management of psychological stress.

### Interreality as an Effective Clinical Protocol for Stress Management

Although different studies in the past may have evaluated the use of VR in the treatment of posttraumatic stress disorder, this is, to our knowledge, the first randomized controlled trial evaluating a technology-enhanced treatment program with active therapeutic involvement for the management of psychological stress.

The main issue in dealing with stress is that it is very personal. Thus, stress-related disorders cannot be explained simply on the basis of the adverse situations experienced by people. These disorders depend a great deal on how the person experiencing a stressor is put together psychologically and physically. So the focus for assessment, prediction, and treatment should be the situated experience of the person. And this is difficult to achieve using both CBT and the available VR protocols. The emotions and problems experienced by the patient in the real world are not directly assessed and/or addressed in real time by CBT; VR is a “closed” experience, separate from the emotions and behaviors experienced by the patient in the real world.

In this study, we provided initial evidence that a possible approach to overcome these issues is the use of the “Interreality” paradigm, which combines assessment and treatment within a hybrid environment, bridging physical and virtual worlds [40-42]. Specifically, the proposed protocol bridged experiential virtual scenarios (fully controlled by the therapist, used to learn coping skills and improve self-efficacy) with real-time monitoring and support (identifying critical situations and assessing clinical change), using advanced technologies (virtual worlds, wearable biosensors, and smartphones).

Possible explanations for the higher efficacy of the proposed approach are:

1. The added value offered to individuals by VR over guided imagery for the acquisition of behavioral and coping skills: As the findings indicate, VR is able to help individuals obtain a marked increase in the relaxation levels early in the therapy (from Session 3), while guided imagery is able to obtain the same relaxation levels only later on (from Session 7).
2. The added value offered to the individuals by real-time stress monitoring and support over traditional diary reporting: As suggested by qualitative reports, the IR sample strongly appreciated the possibility of having real-time stress monitoring and, if needed, use of the smartphone to experience the same relaxation protocols learned in the therapist’s office in real-life settings.
3. The added value offered to the therapists by real-time stress monitoring over traditional diary reporting: In the early part of each session, IR therapists used the recorded real-time stress data to identify critical situations to be addressed in virtual scenarios. CBT therapists were not able to obtain similar detailed information from the diaries compiled by their subjects.

### Strengths and Limitations

This study had several strengths as a direct test of the effects of an IR technology-enhanced protocol in the context of psychological stress management. The first strength of this study is that IR was compared with the best validated approach for stress management, CBT, as identified by the Cochrane Database of Systematic Reviews [36-38], demonstrating the added value offered by IR technology for psychological stress problems. Another important strength is the use, as primary outcome variables, of reliable theory-based measures of situational state and chronic trait anxiety. The third strength of IR is the use of VR to enhance the efficacy of biofeedback intervention. Biofeedback training is regarded as a useful technique to reduce anxiety symptoms (eg, [89]). The most common limitation of biofeedback and relaxation training is that it requires time commitment and implementation effort on behalf of the patient, who can rely only on very simple audio and visual cues provided by the system to learn about body responses to stress. In VR-based biofeedback, elements of the virtual environment are directly modified by the patient’s physiological parameters recorded in real time (eg, in the “Campfire” scenario, physiological parameters control the fire intensity, so that the reduction of the patient’s physiological activation results in a reduction of the fire until it goes out). Thus, patients receives immediate feedback on their level of activation (as in the traditional biofeedback techniques), but with richer and more engaging 3D visual cues [90]. Unfortunately, our experimental design does not allow determination of the relative benefits of VR experiences and VR-based biofeedback intervention in reducing stress and anxiety, which is a future research goal.

However, the present findings should not be considered definitive. First, the study did not include a follow-up assessment of behavior maintenance in the long term. We have planned a follow-up research study to assess the participant samples again at 6-month and 12-month intervals.

A further limitation is that the study did not include any measure of physiological stress (eg, cortisol levels in blood, saliva, or urine samples). However, we justified this choice in the Methods following the results of a recent study demonstrating the low sensitivity of cortisol levels for individuals under long-term stress exposure [48].

Third, while our study design provided a strong test of the efficacy of the IR protocol, it did not allow an evaluation of the specific effectiveness of the different technological tools included in it. Further research is needed to identify the effective elements of the IR protocol and the optimal amount of technological intervention needed. As underlined clearly by qualitative reports, the usability, invasiveness, and complexity of the provided technology are the main barriers for a wide use of the proposed protocol: 2 out of 3 teachers and 1 out of 4 nurses involved in the EG evaluated as “high” or “very high” the amount of technological effort required by the protocol. In particular, the main issues indicated by the participants are the duration/charging of the batteries for both smartphone and biosensors, the Bluetooth pairing of the smartphone with the biosensors, the invasiveness of the biosensors, and the difficulty in consulting real-time stress data in some working situations (eg, teaching for teachers, consulting for nurses).

Finally, the IR technology–enhanced treatment is more expensive for both therapists (VR hardware including computer: €2200; biosensors: €2500) and patients (smartphone: €650; biosensor: €1000). Nevertheless, the rapid technological development is reducing the cost of the required technology. For example, at the end of the trial, the cost of a comparable body-worn wireless sensor decreased from €1000 (Empatica E3 Wrist Sensor) to €150 (Angel Health Monitor).

### Conclusions

Our findings provide initial evidence that a technology-enhanced IR protocol provides better outcomes than the traditionally accepted gold standard for psychological stress treatment, CBT. However, a follow-up study assessing behavior maintenance in the long term is needed in order to provide additional evidence of the effectiveness of IR. In particular, unlike CBT, IR was able to significantly reduce chronic trait anxiety and better improve emotion-related coping skills. Consequently, these findings constitute a sound foundation and rationale for future research in technology-enhanced protocols for psychological stress management. In particular, our study provides the evidence required to justify carrying out much larger follow-up trials to identify the most effective technological interventions and the optimal amount of technological support needed.

## Multimedia Appendix 1

CONSORT-EHEALTH Checklist V1.6.2 [43].

## Multimedia Appendix 2

Stressful scenarios for the project.

## Multimedia Appendix 3

EG and CG study protocols.

## Abbreviations

ANCOVA: analysis of covariance
CBT: cognitive behavioral therapy
CG: control group
COPE: Coping Orientation to Problems Experienced inventory
COPE-NIV: Coping Orientation to the Problems Experienced—New Italian Version
DSM-IV: Diagnostic and Statistical Manual of Mental Disorders, 4th edition
EG: experimental group
IR: Interreality
MINI: Mini-International Neuropsychiatric Interview
PSM: Psychological Stress Measure
PSS: Perceived Stress Scale
PTSD: posttraumatic stress disorders
STAI: State-Trait Anxiety Inventory
SWLS: Satisfaction With Life Scale
WL: wait-list group
VAS-A: Visual Analogue Scale for Anxiety
VR: virtual reality
