# Supplementary material for: Experiential Virtual Scenarios With Real-Time Monitoring (Interreality) for the Management of Psychological Stress: A Block Randomized Controlled Trial
Source: J Med Internet Res. 2014 Jul 8;16(7):e167. doi: 10.2196/jmir.3235 (PMC4115267; doi:10.2196/jmir.3235)
Supplement: Supplementary file 1 [file jmir_v16i7e167_app1.pdf]

CONSORT-EHEALTH Checklist V1.6.2 Report

(based on CONSORT-EHEALTH V1.6), available at [http://tinyurl.com/consort-ehealth-v1-6].

3235

Date completed

1/5/2014 14:37:48

by

Andrea Gaggioli

Interreality for the Management of Psychological Stress: a Block Randomized Controlled Trial

TITLE

1a-i) Identify the mode of delivery in the title

"Interreality for the Management of Psychological Stress"

1a-ii) Non-web-based components or important co-interventions in title

"Interreality for the Management of Psychological Stress"

1a-iii) Primary condition or target group in the title

"Interreality for the Management of Psychological Stress"

ABSTRACT

1b-i) Key features/functionalities/components of the intervention and comparator in the METHODS section of the ABSTRACT

"To evaluate the efficacy of a new technological paradigm – Interreality –for the management and training of psychological stress. The main feature of Interreality is a twofold link between the virtual and the real world achieved through experiential virtual scenarios (fully controlled by the therapist, used to learn coping skills and improve self-efficacy) with real time monitoring and support (identifing critical situations and assessing clinical change) using advanced technologies (virtual worlds, wearable biosensors and smartphones)."

1b-ii) Level of human involvement in the METHODS section of the ABSTRACT

"The main feature of Interreality is a twofold link between the virtual and the real world achieved through experiential virtual scenarios (fully controlled by the therapist, used to learn coping skills and improve self-efficacy) with real time monitoring and support (identifing critical situations and assessing clinical change) using advanced technologies (virtual worlds, wearable biosensors and smartphones)."

1b-iii) Open vs. closed, web-based (self-assessment) vs. face-to-face assessments in the METHODS section of the ABSTRACT

"The following questionnaires were administered offline under therapist's supervision to each participant at pre-treatment, and upon completion of the trial (after 5 weeks):"

1b-iv) RESULTS section in abstract must contain use data

"Participants within each block were randomly assigned to the: (1) Experimental Group (EG: N=40; B1=20, B2=20): received a 5-week treatment based on the Interreality paradigm; (2) Control Group (CG: N=42; B1=22, B2=20): received a 5-week traditional stress management CBT-based training; (3) the Wait List group (WL: N=39, B1=19, B2=20): reassessed and compared with the two other groups five weeks after the initial evaluation."

1b-v) CONCLUSIONS/DISCUSSION in abstract for negative trials

Not applicable: It is a positive trial

INTRODUCTION

2a-i) Problem and the type of system/solution

"Starting from the above premises, the main goal of this study are:
- to define and develop an Interreality protocol for the management of psychological stress;
- to compare, within a controlled study, its efficacy with a comparable protocol based on the CBT approach.
We hypothesize that the Interreality protocol is more effective than both standard CBT and a wait list condition in: 1- reducing the level of chronic "trait" stress; 2- reducing the perceived stress and quality of life; 3- improving the coping skills of the individual."

2a-ii) Scientific background, rationale: What is known about the (type of) system

"The trouble with managing psychological stress is that it is very personal. So the focus for assessment, prediction and treatment has to be the situated experience of the individual. And this is difficult to achieve using the available VR protocols for PTSD: from a clinical viewpoint, in these protocols VR is a “closed” experience, separated from the emotions and behaviors experienced by the patient in the real world. In other words, VR exposure tries to change cognitive content per se, rather than changing the context in which cognitions are experienced [30]: the behavior of the patient in VR has no direct effects on the real life experience; the emotions and problems experienced by the patient in the real world are not directly addressed in the VR exposure. More, it focuses on patients' thoughts and behaviours but does not address social support and coping skills.
To overcome these issues Riva and colleagues suggested the use of the “Interreality” paradigm that integrates assessment and treatment within a hybrid environment, bridging physical and virtual world [31-33]: (a) behaviour in physical world influences the experience in the virtual one; (b) behaviour in the virtual world influences the experience in the real one. Specifically, they suggest that bridging experiential virtual scenarios (fully controlled by the therapist, used to learn healthy behaviors and coping skills) with real experiences (by monitoring in real time stress levels and providing situated support) – using advanced technologies (virtual worlds, wearable biosensors and a smartphone) is a feasible way to address the complexity of psychological stress (see Figure 1)."

METHODS

3a) CONSORT: Description of trial design (such as parallel, factorial) including allocation ratio

"The study is designed as a multicentric randomized block controlled trial involving participants recruited from two different worker populations - teachers and nurses - which are highly exposed to psychological stress (the Consort Flow Chart is reported in Figure 3). "

3b) CONSORT: Important changes to methods after trial commencement (such as eligibility criteria), with reasons

No significant changes to methods

3b-i) Bug fixes, Downtimes, Content Changes

"As underlined clearly by qualitative reports, the usability, invasiveness and complexity of the provided technology are the main barriers for a wide use of the proposed protocol: two out of three teachers and one out of four nurses involved in the EG group evaluated as “high” or “very high” the amount of technological effort required by the protocol. In particular, the main issues indicated by the participants are the duration/charging of the batteries for both smartphone and biosensors, the bluetooth pairing of the smartphone with the biosensors, the invasiveness of the biosensors and the difficulty in consulting real time stress data in some working situations (e.g. teaching for teachers, consulting for nurses)."

4a) CONSORT: Eligibility criteria for participants

"Criteria for participation included the following: (1) A high level of perceived stress (>= 7) as measured on a 10-item visual-analogue scale; (2) A high level of relevance of stress for personal health (>= 7) as measured on a 10-item visual-analogue scale; (3) A low level of self-efficacy related to stress management (<= 5) as measured on a 10-item visual-analogue scale; (4) No DSM_IV-TR Axis I disorders; (5) Age between 25 and 60 years; (6) No psychotherapy received for their psychological stress; (7) No pharmacotherapy; (8) No history of neurological diseases, psychosis, alcohol or drug dependence; (9) No migraine, headache, or vestibular abnormalities. Both males and females are included. "

4a-i) Computer / Internet literacy

"We also assessed computer literacy at the start of the trial but no significant differences were found between groups."

4a-ii) Open vs. closed, web-based vs. face-to-face assessments:

"The following questionnaires were administered offline under therapist's supervision to each participant at pre-treatment, and upon completion of the trial (after 5 weeks):<br>•State-Trait Anxiety Inventory Form Y-2 (STAI) [53, 54]. STAI consists of two scales containing 20 items each that measure anxiety in adults. The STAI clearly differentiates between the temporary condition of "state anxiety" (STAI Form Y-1, also known as STAI-Y1) and the more general and long-standing quality of "trait anxiety."(STAI Form Y-2, also known as STAI-Y2). For the initial and the final assessment in the trial we used the STAI Y-2, i.e. the Trait version of the STAI, which measures characteristic tendencies to be anxious.<br>•Coping Orientation to Problems Experienced inventory (COPE) [55, 56]. COPE Inventory was developed to assess a broad range of coping responses. It presents fourteen scales all assessing different coping dimensions such, as an example, active coping, planning, and using instrumental support. Each scale contains two items, for a total of 60 items altogether. This inventory can be used to assess trait coping (the usual way people cope with stress in everyday life) and state coping (the particular way people cope with a specific stressful situation).<br>•Perceived Stress Scale (PSS) [57]. The PSS is a 10-item self-reported measure designed to deal with the degree to which situations in an individual's life are appraised as stressful. It was originally developed as a 14-item scale that assessed the perception of stressful experiences over the previous month using a five-point Likert scale. Later, the authors reported that the 10-item version (PSS-10) showed stronger psychometric characteristics in comparison to the 14-item scale (Cohen & Williamson, 1988).<br>•Psychological Stress Measure (PSM) [58, 59]. The questionnaire consists of 49 items, based on the various individual perceptions of cognitive, physiological, and behavioral state of subjects. PSM provides a global score of stress and some partial sub-scores. The individual is asked to answer on the basis of how he or she has been feeling in the last 4-5 days. The global score of the PSM is compared with ground truth scores, which give threshold cut off on the basis of the gender (103 for male and 110 for female subjects).<br>•Satisfaction with Life Scale (SWLS) [60, 61]. The SWLS is a measure of life satisfaction (subjective well-being). The 5-item questionnaire is designed to measure global cognitive judgments of satisfaction with one`s own life.<br>Secondary Outcome Measures<br>Individuals were also assessed at the beginning and at the end of each of the 8 training sessions using the following questionnaires:<br>•State-Trait Anxiety Inventory Form Y-1 (STAI Y-1) [53, 54]. STAI Y-1 addresses state anxiety, which could be defined as a temporary emotional condition characterized by apprehension, tension, and fear about a particular situation or activity. This inventory consists a of a 20 item scale, like the STAI Y-2.<br>•Visual Analogue Scale for Anxiety (VAS-A) is an instrument that measure anxiety across a continuum. It is a horizontal line, 100 millimeter in length, anchored by word descriptors at each end (No anxiety; Very severe anxiety). Individuals mark on the line the point that they feel represents their perception of their own current state. The VAS-A score is determined by measuring in millimeters from the left hand end of the line to the point that the person marks."
**4a-iii) Information giving during recruitment**
"All the participants signed an informed consent form. The consent form explained the goal of the treatment, its duration, the involvement of the patients and, for EG individuals, the possible side effects related to the extended use of immersive virtual environments: ocular problems, disorientation and balance disturbances, and nausea."
**4b) CONSORT: Settings and locations where the data were collected**
"Treatment block for teachers (Block 1: N=61) was offered by therapists from Istituto Auxologico Italiano both in the Istituto Scientifico Ospedale San Luca, Milano, Italy and in the schools where teachers worked. Treatment block for nurses (Block 2: N=60) was offered by therapists from National Research Council of Italy (CNR) in the Institute of Clinical Physiology (IFC), Messina, Italy"
**4b-i) Report if outcomes were (self-)assessed through online questionnaires**
Only offline questionnaires were used in the trial
**4b-ii) Report how institutional affiliations are displayed**
Not relevant
**5) CONSORT: Describe the interventions for each group with sufficient details to allow replication, including how and when they were actually administered**
**5-i) Mention names, credential, affiliations of the developers, sponsors, and owners**

"RG, GAn, PF, RS, GAl, WB and PG contributed to the design of the study. RG prepared the initial drafts of the paper. GAn, PF, SS and WB critically reviewed the initial drafts of the paper. PG, BG, TG, and VC developed the different technologies used in the studies. PF, ML, BM, RS, BM and CG were involved in the treatment and collected the data. CP, SS, PF, and RG carried out the analysis and interpretation of data. All authors contributed to the final draft of the paper and approved the final version for publication. All authors report no conflicting interests. This study was funded by European Union funded Interstress project (ICT Grant Number FP7-247685)."
**5-ii) Describe the history/development process**
The most critical technologies used in the trial - virtual reality and decision support system - were developed and validated in previous studies:<br><br>40.Riva G, Gaggioli A, Grassi A, Raspelli S, Cipresso P, Pallavicini F, et al. NeuroVR 2--a free virtual reality platform for the assessment and treatment in behavioral health care. Studies in health technology and informatics 2011;163:493-495.<br>41.Riva G, Gaggioli A, Villani D, Preziosa A, Morganti F, Corsi R, et al. NeuroVR: an open source virtual reality plat- form for clinical psychology and behavioral neurosciences. Studies in health technology and informatics 2007;125:394-399.<br>42.Riva G, Gaggioli A, Villani D, Preziosa A, Morganti F, Corsi R, et al. NeuroVR: an open source virtual reality platform for clinical psychology and behavioral neurosciences. Studies in Health Technology and Informatics 2007;125:394-399.<br>47.Manzoni GM GA, Preziosa A, Pagnini F, Castelnuovo G, Molinari E, Riva G. New Technologies and Relaxation: An exploratory study on obese patients with emotional eating. The Role Of Presence. Journal of Cybertherapy and Rehabilitation 2008;1:182-192.<br>48.Pallavicini F, Algeri D, Repetto C, Gorini A, Riva G. Biofeedback, virtual reality and mobile phones in the treatment of Generalized Anxiety Disorder (GAD): A phase-2 controlled clinical trial. Journal of Cybertherapy and Rehabiliation 2009;2:315-327.<br>49.Gaggioli A, Pioggia G, Tartarisco G, Baldus G, Ferro M, Cipresso P, et al. A system for automatic detection of momentary stress in naturalistic settings. Stud Health Technol Inform 2012;181:182-186.<br>50.Tartarisco G, Baldus G, Corda D, Raso R, Arnao A, Ferro M, et al. Personal Health System architecture for stress monitoring and support to clinical decisions. Computer Communication 2012;35:1296-1305.
**5-iii) Revisions and updating**
Development and/or content was “frozen” during the trial with technological interventions only if needed.
**5-iv) Quality assurance methods**
We followed the basic guidelines provided her by FDA:<br><br>Meeker-O’Connell A. Enhancing clinical trial quality: CDER perspective. [Last accessed on 2011 May 2]. Available from: http: / / www.fdanews.com / ext/ files / Conference / FIS10Presentations / MeekerOConnell-HarmonizingRegulatoryApproaches.pdf .<br><br>The FDA recommended a four-step systems approach, as follows:<br><br>Say what you do<br>Do what you say<br>Prove it<br>Improve it (if needed)
**5-v) Ensure replicability by publishing the source code, and/or providing screenshots/screen-capture video, and/or providing flowcharts of the algorithms used**

The NeuroVR 2 software used to provide virtual reality interventions is available for free at the web address: www.neurovr.org
The description of the Interreality paradigm is fully explained in the paper:

"The experimental group used an Interreality protocol based on the following technologies (see Figure 4 for details):

Figure 4: The technologies used by the Experimental Group

o3D Virtual Scenarios (in the therapist's office):

•immersive role-playing scenarios in which the individual interacts with potentially stressful experiences: According to a literature review and to the results of a qualitative analysis, different virtual stressful scenarios were generated for both teachers and nurses (see Table 2). The stressful scenarios were played by real actors and included into the virtual environments (using the free virtual reality platform NeuroVR 2 [40-42]) after a video post-production.
•immersive natural scenarios used to learn specific relaxation techniques: In the last years, VR has been used in different clinical protocols to facilitate relaxation processes in stressed or anxious subjects [43-45] by visually presenting relaxing scenes. The Interreality relaxation environments were created on the basis of similar virtual relaxing environments validated in previous studies [44, 46-48] and they include a beach, a lake with a waterfall, a campfire in a mountain resort, and a desert oasis.

Table 2: The different virtual stressful scenarios

Virtual scenarios for teachersVirtual scenarios for nurses
1.Workload;
2.Class management;
3.Coping with parent's criticism;
4.Relationship with boss;
5.Coping with parent's handling efforts;
6.Relationship with co-workers;
7.Conflict management.1.Managing the patients' relatives;
2.Managing patients' complaints;
3.Managing a medical emergency situation;
4.Relationship with colleagues;
5.Managing relatives'/caregivers anxiety;
6.Distribution of work tasks;
7.Patient-doctor communication;
8.Managing patient's anxiety;
9.Unsuccessful collaboration/communication with colleagues;
10.Discussions among medical doctors.

oBiosensors (Personal Biomonitoring System):

•In the therapist's office (Biofeedback): typically 3D virtual worlds are closed experience and do not reflect in any way the real activity and status of the users. In Interreality, instead, physiological sensors (heart rate and heart rate variability) are used to track the emotional/health status of the individual and to influence his/her experience in the virtual world. Specifically, to improve the efficacy of the relaxing environment, some features of the experience

– e.g. the size of the fire, or the waterfall flow rate – were driven by the emotional status of the patient as measured by biosensors (heart rate or heart rate variability).
•Outside therapist's office (Physiological Data Recording): To assess the level of contextual stress each individual was provided a body-worn wireless sensor (EMPATICA E3 wrist sensor) that was able to record and transmit psychophysiological (HR and HRV) and activity data in real-time.

oSmartphone (Outside therapist's office):
•Stress Tracking: the data received from the wireless sensor are assessed in real time by a decision support system (a the description of the used system can be found in [49, 50]). This system provides the user with a graphic representation of the current stress level experi enced, and allows to check the history of stress levels variations experienced at different timescale (e.g., day, week, month).
•Contextualized Homeworks: according to the performance achieved in the therapist's office and to level of stress assessed by the decision support system the individual was able to experience on the smartphone different guided relaxation and biofeedback virtual experiences (non immersive) similar to the ones experienced in the therapist's office."

5-vi) Digital preservation

The NeuroVR 2 software used to provide virtual reality interventions is available for free at the web address: www.neurovr.org

5-vii) Access

"All the above technology, including smartphone and biosensors, was lended for free at the EG participants. More, all the required data connection fees for real time stress monitoring were paid by the trial. "

5-viii) Mode of delivery, features/functionalities/components of the intervention and comparator, and the theoretical framework

"Specifically, the protocol of the experimental condition had the following schema:

oAssessment Session (Session 1): The session started with a discussion with the clinician about the assessment week. Then, after a brief introduction to the specific content of this session, the psychometric questionnaires were administered for the first time (see below) and the physiological baseline of the participant was recorded for three minutes. To measure the psychological variations occurring during the different stressful virtual scenarios, subjects completed the VAS-A and the STAI-Y1 at the baseline and after each scenario. During stressful exposition participant physiological parameters were also recorded. Besides the stressful scenarios (see Table 2) each participant was assessed in a neutral virtual environment and in one in which he or she completed a cognitive task in front of a virtual audience. These situations were used to identify the situations inducing the highest level of stress. At the end of this session, the clinician explained to the participant how to use the smartphone and the body-worn wireless sensor.
oTraining Session (Session 2-9): The following sessions were dedicated to teach participants to cope with stress, through cognitive restructuring techniques and the teaching of relaxation exercises. Each session lasted about one hour and was divided into four parts: homework checking, exposure to a stressful, relaxation exercises (with or without biofeedback) and homework assignment. The clinician decided with the participant the specific stress scenarios to work on in the exposure, working on it (cognitive restructuring) with the clinician during at least two consecutive sessions. Cognitive restructuring was used to help patients to identify and challenge their erroneous beliefs and interpretations. Specifically, patients were taught to look at their negative beliefs, look for possible alternative explanations and ways of thinkin, and evaluate the pros and cons of maintaining them. Relaxation was induced through the immersion in a natural scenario selected by the subject in which he/she could move and interact. The experience was customized with different pre-recorded audio narratives describing the specific setting and guiding the execution of different progressive muscle relaxation relaxation/deep breathing exercises. More, the scenario was experienced with or without VR biofeedback, in alternate way during sessions. At the end of the sessions, the clinician explained to the participant how to use the smartphone and the body-worn wireless sensor to check the level of contextual stress and to carry out the contextualized homeworks.
oFollow up Session (Session 10): To verify the efficacy of the training, during the final session participants were reassessed through the administration of psychometric questionnaires. Moreover participants were exposed again to the different stressful virtual scenarios, following the same procedure of the assessment session (session 1). At the end of this assessment, the clinician discussed with the participant about the protocol and its perceived efficacy/usefulness."

5-ix) Describe use parameters

"Specifically, the protocol of the experimental condition had the following schema:<br><br>oAssessment Session (Session 1): The session started with a discussion with the clinician about the assessment week. Then, after a brief introduction to the specific content of this session, the psychometric questionnaires were administered for the first time (see below) and the physiological baseline of the participant was recorded for three minutes. To measure the psychological variations occurring during the different stressful virtual scenarios, subjects completed the VAS-A and the STAI-Y1 at the baseline and after each scenario. During stressful exposition participant physiological parameters were also recorded. Besides the stressful scenarios (see Table 2) each participant was assessed in a neutral virtual environment and in one in which he or she completed a cognitive task in front of a virtual audience. These situations were used to identify the situations inducing the highest level of stress. At the end of this session, the clinician explained to the participant how to use the smartphone and the body-worn wireless sensor.<br>oTraining Session (Session 2-9): The following sessions were dedicated to teach participants to cope with stress, through cognitive restructuring techniques and the teaching of relaxation exercises. Each session lasted about one hour and was divided into four parts: homework checking, exposure to a stressful, relaxation exercises (with or without biofeedback) and homework assignment. The clinician decided with the participant the specific stress scenarios to work on in the exposure, working on it (cognitive restructuring) with the clinician during at least two consecutive sessions. Cognitive restructuring was used to help patients to identify and challenge their erroneous beliefs and interpretations. Specifically, patients were taught to look at their negative beliefs, look for possible alternative explanations and ways of thinkin, and evaluate the pros and cons of maintaining them. Relaxation was induced through the immersion in a natural scenario selected by the subject in which he/she could move and interact. The experience was customized with different pre-recorded audio narratives describing the specific setting and guiding the execution of different progressive muscle relaxation relaxation/deep breathing exercises. More, the scenario was experienced with or without VR biofeedback, in alternate way during sessions. At the end of the sessions, the clinician explained to the participant how to use the smartphone and the body-worn wireless sensor to check the level of contextual stress and to carry out the contextualized homeworks.<br>oFollow up Session (Session 10): To verify the efficacy of the training, during the final session participants were reassessed through the administration of psychometric questionnaires. Moreover participants were exposed again to the different stressful virtual scenarios, following the same procedure of the assessment session (session 1). At the end of this assessment, the clinician discussed with the participant about the protocol and its perceived efficacy/usefulness."
**5-x) Clarify the level of human involvement**

"Specifically, the protocol of the experimental condition had the following schema:<br><br>oAssessment Session (Session 1): The session started with a discussion with the clinician about the assessment week. Then, after a brief introduction to the specific content of this session, the psychometric questionnaires were administered for the first time (see below) and the physiological baseline of the participant was recorded for three minutes. To measure the psychological variations occurring during the different stressful virtual scenarios, subjects completed the VAS-A and the STAI-Y1 at the baseline and after each scenario. During stressful exposition participant physiological parameters were also recorded. Besides the stressful scenarios (see Table 2) each participant was assessed in a neutral virtual environment and in one in which he or she completed a cognitive task in front of a virtual audience. These situations were used to identify the situations inducing the highest level of stress. At the end of this session, the clinician explained to the participant how to use the smartphone and the body-worn wireless sensor.<br>oTraining Session (Session 2-9): The following sessions were dedicated to teach participants to cope with stress, through cognitive restructuring techniques and the teaching of relaxation exercises. Each session lasted about one hour and was divided into four parts: homework checking, exposure to a stressful, relaxation exercises (with or without biofeedback) and homework assignment. The clinician decided with the participant the specific stress scenarios to work on in the exposure, working on it (cognitive restructuring) with the clinician during at least two consecutive sessions. Cognitive restructuring was used to help patients to identify and challenge their erroneous beliefs and interpretations. Specifically, patients were taught to look at their negative beliefs, look for possible alternative explanations and ways of thinkin, and evaluate the pros and cons of maintaining them. Relaxation was induced through the immersion in a natural scenario selected by the subject in which he/she could move and interact. The experience was customized with different pre-recorded audio narratives describing the specific setting and guiding the execution of different progressive muscle relaxation relaxation/deep breathing exercises. More, the scenario was experienced with or without VR biofeedback, in alternate way during sessions. At the end of the sessions, the clinician explained to the participant how to use the smartphone and the body-worn wireless sensor to check the level of contextual stress and to carry out the contextualized homeworks.<br>oFollow up Session (Session 10): To verify the efficacy of the training, during the final session participants were reassessed through the administration of psychometric questionnaires. Moreover participants were exposed again to the different stressful virtual scenarios, following the same procedure of the assessment session (session 1). At the end of this assessment, the clinician discussed with the participant about the protocol and its perceived efficacy/usefulness."
**5-xi) Report any prompts/reminders used**
No prompts or remainder were offered to the users if not required by them. Individuals in the EG were able to receive messages on their smartphone, if set up by them, at specific stress thresholds.
**5-xii) Describe any co-interventions (incl. training/support)**
See 5-x, more: "All the participants received a one-week group technology training plus personalized support sessions if needed."
**6a) CONSORT: Completely defined pre-specified primary and secondary outcome measures, including how and when they were assessed**

"Primary Outcome Measures<br>The following questionnaires were administered offline under therapist's supervision to each participant at pre-treatment, and upon completion of the trial (after 5 weeks):<br>•State-Trait Anxiety Inventory Form Y-2 (STAI) [53, 54]. STAI consists of two scales containing 20 items each that measure anxiety in adults. The STAI clearly differentiates between the temporary condition of "state anxiety" (STAI Form Y-1, also known as STAI-Y1) and the more general and long-standing quality of "trait anxiety."(STAI Form Y-2, also known as STAI-Y2). For the initial and the final assessment in the trial we used the STAI Y-2, i.e. the Trait version of the STAI, which measures characteristic tendencies to be anxious.<br>•Coping Orientation to Problems Experienced inventory (COPE) [55, 56]. COPE Inventory was developed to assess a broad range of coping responses. It presents fourteen scales all assessing different coping dimensions such, as an example, active coping, planning, and using instrumental support. Each scale contains two items, for a total of 60 items altogether. This inventory can be used to assess trait coping (the usual way people cope with stress in everyday life) and state coping (the particular way people cope with a specific stressful situation).<br>•Perceived Stress Scale (PSS) [57]. The PSS is a 10-item self-reported measure designed to deal with the degree to which situations in an individual's life are appraised as stressful. It was originally developed as a 14-item scale that assessed the perception of stressful experiences over the previous month using a five-point Likert scale. Later, the authors reported that the 10-item version (PSS-10) showed stronger psychometric characteristics in comparison to the 14-item scale (Cohen & Williamson, 1988).<br>•Psychological Stress Measure (PSM) [58, 59]. The questionnaire consists of 49 items, based on the various individual perceptions of cognitive, physiological, and behavioral state of subjects. PSM provides a global score of stress and some partial sub-scores. The individual is asked to answer on the basis of how he or she has been feeling in the last 4-5 days. The global score of the PSM is compared with ground truth scores, which give threshold cut off on the basis of the gender (103 for male and 110 for female subjects).<br>•Satisfaction with Life Scale (SWLS) [60, 61]. The SWLS is a measure of life satisfaction (subjective well-being). The 5-item questionnaire is designed to measure global cognitive judgments of satisfaction with one`s own life.<br>Secondary Outcome Measures<br>Individuals were also assessed at the beginning and at the end of each of the 8 training sessions using the following questionnaires:<br>•State-Trait Anxiety Inventory Form Y-1 (STAI Y-1) [53, 54]. STAI Y-1 addresses state anxiety, which could be defined as a temporary emotional condition characterized by apprehension, tension, and fear about a particular situation or activity. This inventory consists a of a 20 item scale, like the STAI Y-2.<br>•Visual Analogue Scale for Anxiety (VAS-A) is an instrument that measure anxiety across a continuum. It is a horizontal line, 100 millimeter in length, anchored by word descriptors at each end (No anxiety; Very severe anxiety). Individuals mark on the line the point that they feel represents their perception of their own current state. The VAS-A score is determined by measuring in millimeters from the left hand end of the line to the point that the person marks.<br>"
**6a-i) Online questionnaires: describe if they were validated for online use and apply CHERRIES items to describe how the questionnaires were designed/deployed**
No online questionnaires were used
**6a-ii) Describe whether and how "use" (including intensity of use/dosage) was defined/measured/monitored**
All treatments included 10 sessions. We did not plan any use tracking outside the therapist's office.
**6a-iii) Describe whether, how, and when qualitative feedback from participants was obtained**
"Qualitative feedback was also obtained using a semi-structured questionnaire at pre-treatment, and upon completion of the trial."
**6b) CONSORT: Any changes to trial outcomes after the trial commenced, with reasons**
None
**7a) CONSORT: How sample size was determined**
**7a-i) Describe whether and how expected attrition was taken into account when calculating the sample size**
"By assessing on a preliminary sample (N=15) the effect size (0.27223) of the treatment using the STAI Y2 score (level of chronic "trait" stress) as primary outcome variable, we found that a sample size of 30 participants for group was enough to achieve a power of 0.82. Using the trial sample size of 40 participants we achieved a power of more than 0.9."

**7b) CONSORT: When applicable, explanation of any interim analyses and stopping guidelines**
None
**8a) CONSORT: Method used to generate the random allocation sequence**
The http://www.randomizer.org web site.
**8b) CONSORT: Type of randomisation; details of any restriction (such as blocking and block size)**
"The study is designed as a multicentric randomized block controlled trial involving participants recruited from two different worker populations - teachers and nurses - which are highly exposed to psychological stress (the Consort Flow Chart is reported in Figure 3)."
**9) CONSORT: Mechanism used to implement the random allocation sequence (such as sequentially numbered containers), describing any steps taken to conceal the sequence until interventions were assigned**
http://www.randomizer.org
**10) CONSORT: Who generated the random allocation sequence, who enrolled participants, and who assigned participants to interventions**
http://www.randomizer.org
**11a) CONSORT: Blinding - If done, who was blinded after assignment to interventions (for example, participants, care providers, those assessing outcomes) and how**
**11a-i) Specify who was blinded, and who wasn't**
Not applicable
**11a-ii) Discuss e.g., whether participants knew which intervention was the "intervention of interest" and which one was the "comparator"**
Not applicable
**11b) CONSORT: If relevant, description of the similarity of interventions**
Not applicable
**12a) CONSORT: Statistical methods used to compare groups for primary and secondary outcomes**
"Our primary end points are the change of the level of chronic "trait" stress, perceived stress and coping skills from baseline (pre) to the end of intervention (post). Secondary outcomes included the change in situational "state" stress scores from start (pre) to the end of intervention (post) in each treatment session.<br>For primary analyses, stress and coping scores were assessed by ANCOVA, with post-treatment scores as the baseline variable, while the pre-treatment scores served as covariates. This approach allowed us to "adjusts" posttest scores for the variability on the pretest ones produced by the block design [62]. A two-sided P value of 0.05 or less was considered be statistically significant.<br>For secondary analyses, the experimental and control groups were simultaneously taken into the analysis of variance model for repeated measures (T=8).<br>Differential effects of the treatments were determined using post-hoc analyses. In particular, to reduce the risk of type I errors, we used the Bonferroni post-hoc procedure with an adjusted Experimentwise Error Rate (EER): 0.05 for each variable in a three-group analysis and 0.025 for each variable in a four-group analysis [63].<br>Prior to analysis, the distributions for the outcome variables were examined. We detected univariate outliers using box plots."
**12a-i) Imputation techniques to deal with attrition / missing values**
No participants dropped out of the trial
**12b) CONSORT: Methods for additional analyses, such as subgroup analyses and adjusted analyses**
Not applicable
RESULTS
**13a) CONSORT: For each group, the numbers of participants who were randomly assigned, received intended treatment, and were analysed for the primary outcome**

"A sample of high school teachers recruited in Milan (Block 1: R=95) and a sample of pediatric nurses recruited in Messina, Italy (Block 2: R=88) were seen for screening interviews for admission to the study. All patients meeting the inclusion criteria in each block (B1=61, B2= 60) were randomly assigned within the three groups (1) the Experimental Group – EG: N=40, B1=20, B2=20; (2) the Control Group – CG: N=42, B1=22, B2=20; (3) the Wait-List group – WL: N=39, B1=19, B2=20. All the participants signed an informed consent form before entering the study. The sample characteristics are shown in Table 1. £

13b) CONSORT: For each group, losses and exclusions after randomisation, together with reasons

No patient was lost after randomization

13b-i) Attrition diagram

Not applicable

14a) CONSORT: Dates defining the periods of recruitment and follow-up

"Samples were recruited between March 2012 and September 2013."

14a-i) Indicate if critical "secular events" fell into the study period

Not applicable

14b) CONSORT: Why the trial ended or was stopped (early)

Not applicable

15) CONSORT: A table showing baseline demographic and clinical characteristics for each group

The data are included in Table 1

15-i) Report demographics associated with digital divide issues

Critical anagraphic data are included in Table 1

16a) CONSORT: For each group, number of participants (denominator) included in each analysis and whether the analysis was by original assigned groups

16-i) Report multiple "denominators" and provide definitions

Data are reported in Consort Table (figure 1)

16-ii) Primary analysis should be intent-to-treat

We used an intent-to-treat approach but no individuals left the trial after randomization

17a) CONSORT: For each primary and secondary outcome, results for each group, and the estimated effect size and its precision (such as 95% confidence interval)

"The oneway ANCOVA on the pre- post-treatment scores showed a significant group effect (F=4.42; d.f.=2, 107; p<0.014; effect size= 0.74) on the primary outcome variable of chronic "trait " anxiety (STAI Y2). While no significant differences were found in both the Wait List group (WL) and the Control Group (CG) between the pre- and post-measurements, the Experimental Group (EG) was able to obtain a significant anxiety decrease (12%). This datum was confirmed by post-hoc analyses: they revealed significant differences between the EG and the CG (p <0.05), and between the EG and the WL (p <0.01)."

17a-i) Presentation of process outcomes such as metrics of use and intensity of use

"To better understand the differences between the EG and CG we used a repeated measures ANOVA (T=8) to analyze the changes in the situational "state" stress scores (STAI Y1 and VAS-A) before and after each treatment.
The analysis revealed significant TIME (F=3.8; d.f.= 7, 511; p <.01; effect size = .05) and TIME x GROUP (Quadratic contrast: F=4.23; d.f.= 1, 73; p <. 05; effect size = .055) effects in the VAS-A scores. First, both groups progressively increased the relaxation level achieved in a session during the protocol. Second, the the pattern of this increase is curvilinear (i.e. is represented by a curve with one bend in) with a difference between the two groups (see Figure 5): EG showed a marked (>15%) early increase in the relaxation level (from session 3) and a further improvement (>20%) in the next sessions; CG instead showed an initial increase of the relaxation level (>10%) from the first session, with a marked (>15%) increase in the relaxation level only in the last sessions (7-8). The analysis of STAI scores revealed a similar pattern but with a lack of significant values due to the low statistical power (0.23).
We then compared the two blocks (teachers and nurses) to check for differences. As before nurses obtained higher, but not significant, relaxation values than teachers in all the sessions."

**17b) CONSORT: For binary outcomes, presentation of both absolute and relative effect sizes is recommended**
Not applicable
**18) CONSORT: Results of any other analyses performed, including subgroup analyses and adjusted analyses, distinguishing pre-specified from exploratory**
Not applicable
**18-i) Subgroup analysis of comparing only users**
Not applicable
**19) CONSORT: All important harms or unintended effects in each group**
Not applicable
**19-i) Include privacy breaches, technical problems**
Not applicable
**19-ii) Include qualitative feedback from participants or observations from staff/researchers**
"As underlined clearly by qualitative reports, the usability, invasiveness and complexity of the provided technology are the main barriers for a wide use of the proposed protocol: two out of three teachers and one out of four nurses involved in the EG group evaluated as “high” or “very high” the amount of technological effort required by the protocol. In particular, the main issues indicated by the participants are the duration/charging of the batteries for both smartphone and biosensors, the bluetooth pairing of the smartphone with the biosensors, the invasiveness of the biosensors and the difficulty in consulting real time stress data in some working situations (e.g. teaching for teachers, consulting for nurses)."
DISCUSSION
**20) CONSORT: Trial limitations, addressing sources of potential bias, imprecision, multiplicity of analyses**
**20-i) Typical limitations in ehealth trials**
"However, the findings cannot be considered definitive.<br>First, the study did not include a follow-up study to assess behavior maintenance in the long term. We planned a follow-up research to assess again the samples at 6-month and 12-month intervals.<br>Second, the study did not include any measure of physiological stress (e.g., cortisol levels in blood, saliva, or urine samples). We justified this choice in the Methods following the results of a recent study demonstrating the low sensitivity of cortisol levels for individuals under long-term stress exposure [36].<br>Third, while our study design provided a strong test of the efficacy of the IR protocol, it did not allow an evaluation of the effectiveness of the different technological tools included in it."
**21) CONSORT: Generalisability (external validity, applicability) of the trial findings**
**21-i) Generalizability to other populations**
"The first strenght of this study is that IR was compared with the best validated approach for stress management - CBT – as identified by the Cochrane Database of Systematic Reviews [27-29], demonstrating the added value offered by IR technology for psychological stress problems. Another important strenght is the use, as primary outcome variables, of reliable, theory-based measures of situational “state” and chronic “trait” anxiety. However, the findings cannot be considered definitive. "
**21-ii) Discuss if there were elements in the RCT that would be different in a routine application setting**
Not Applicable
**22) CONSORT: Interpretation consistent with results, balancing benefits and harms, and considering other relevant evidence**
**22-i) Restate study questions and summarize the answers suggested by the data, starting with primary outcomes and process outcomes (use)**
"Even if both treatments (Cognitive Behavioral Treatment – CBT and Interreality – IR) were able to significantly reduce perceived stress (with a better outcome for IR) only participants who received IR reported a significant reduction (12% vs 0.5%) in chronic “trait “ anxiety. A similar pattern was found for coping skills: both treatments were able to significantly increase most coping skills but participants who received IR reported a significant higher increase (14% vs 0,3%) in the Emotional Support skill than CBT. In sum, the obtained data demonstrated a better efficacy of IR over CBT in the management of psychological stress."
**22-ii) Highlight unanswered new questions, suggest future research**

"Further research is needed to identify the effective elements of the IR protocol and the optimal amount of technological intervention needed. As underlined clearly by qualitative reports, the usability, invasiveness and complexity of the provided technology are the main barriers for a wide use of the proposed protocol"

Other information

23) CONSORT: Registration number and name of trial registry

ClinicalTrials.gov NCT01683617

24) CONSORT: Where the full trial protocol can be accessed, if available

The protocol was published here: Pallavicini F, Gaggioli A, Raspelli S, Cipresso P, Serino S, Vigna C, et al. Interreality for the management and training of psychological stress: study protocol for a randomized controlled trial. Trials 2013;14:191.

25) CONSORT: Sources of funding and other support (such as supply of drugs), role of funders

This study was funded by European Union funded Interstress project (ICT Grant Number FP7-247685).

X26-i) Comment on ethics committee approval

Not applicable

x26-ii) Outline informed consent procedures

"The study was approved by the Istituto Auxologico Italiano ethical review board in Milan, Italy and conducted according to the 1964 Declaration of Helsinki. All the participants signed an informed consent form. The consent form explained the goal of the treatment, its duration, the involvement of the patients and, for EG individuals, the possible side effects related to the extended use of immersive virtual environments: ocular problems, disorientation and balance disturbances, and nausea."

X26-iii) Safety and security procedures

Not applicable for the general treatment. For virtual reality treatment we checked during every five minutes during immersion the presence of possible side effects. If noticed the therapist stopped the VR session for 5 minutes before starting again. If happening again the VR session was stopped.

X27-i) State the relation of the study team towards the system being evaluated

None relevant
