# Supplementary material for: Experiential Virtual Scenarios With Real-Time Monitoring (Interreality) for the Management of Psychological Stress: A Block Randomized Controlled Trial
Source: J Med Internet Res. 2014 Jul 8;16(7):e167. doi: 10.2196/jmir.3235 (PMC4115267; doi:10.2196/jmir.3235)
Supplement: Supplementary file 3 [file jmir_v16i7e167_app3.pdf]

Multimedia Appendix 3.

Table 1. Study protocol for the experimental group.

Intake session	Introduction of the training	Clinical interview (MINI)	Give STAI Y2, PMS, PSS, COPE and SWLS		Questionnaire about participants' expectations and motivations		Explanation of how to use the PDA and the PMS
Session I<br>Assessment session	Physiological assessment	Give STAI Y1 and VAS-A (baseline)	Exposition to stressful virtual environment n1	Give STAI Y1 and VAS-A (after each scenario)	Repeated for all the 7 stressful environments + neutral one + cognitive task	Exposition to a relaxing virtual environment	Final debriefing
Session II	Preliminary phase	Pre-training	Training			Post-training	Final phase
Training session	Give STAI Y1 and VAS-A	Physiological assessment	Exposition to stressful virtual environment	Cognitive restructuring	Exposition to a relaxing virtual environment	Give STAI Y1 and VAS-A	Delivery of homework and debriefing
Session III	Preliminary phase	Pre-training	Training			Post-training	Final phase
Training session	Give STAI Y1 and VAS-A	Physiological assessment	Exposition to stressful virtual environment	Cognitive restructuring	Biofeedback in virtual reality	Give STAI Y1 and VAS-A	Delivery of homework and debriefing
Session IV	Preliminary phase	Pre-training	Training			Post-training	Final phase
Training session	Give STAI Y1 and VAS-A	Physiological assessment	Exposition to stressful virtual environment	Cognitive restructuring	Exposition to a relaxing virtual environment	Give STAI Y1 and VAS-A	Delivery of homework and debriefing
Session V	Preliminary phase	Pre-training	Training			Post-training	Final phase
Training session	Give STAI Y1 and VAS-A	Physiological assessment	Exposition to stressful virtual	Cognitive restructuring	Biofeedback in virtual reality	Give STAI Y1 and VAS-A	Delivery of homework and

			environment				debriefing
Session VI	Preliminary phase	Pre-training	Training			Post-training	Final phase
Training session	Give STAI Y1 and VAS-A	Physiological assessment	Exposition to stressful virtual environment	Cognitive restructuring	Exposition to a relaxing virtual environment	Give STAI Y1 and VAS-A	Delivery of homework and debriefing
Session VII	Preliminary phase	Pre-training	Training			Post-training	Final phase
Training session	Give STAI Y1 and VAS-A	Physiological assessment	Exposition to stressful virtual environment	Cognitive restructuring	Biofeedback in virtual reality	Give STAI Y1 and VAS-A	Delivery of homework and debriefing
Session VIII	Preliminary phase	Pre-training	Training			Post-training	Final phase
Training session	Give STAI Y1 and VAS-A	Physiological assessment	Exposition to stressful virtual environment	Cognitive restructuring	Exposition to a relaxing virtual environment	Give STAI Y1 and VAS-A	Delivery of homework and debriefing
Session IX	Preliminary phase	Pre-training	Training			Post-training	Final phase
Training session	Give STAI Y1 and VAS-A	Physiological assessment	Exposition to stressful virtual environment	Cognitive restructuring	Biofeedback in virtual reality	Give STAI Y1 and VAS-A	Delivery of homework and debriefing
Session X<br>Final session	Follow up of questionnaires used in the evaluation phase	Physiological assessment	Give STAI Y1 and VAS-A	Exposition to stressful virtual environment n1	Give STAI Y1 and VAS-A	Repeated for all the 7 stressful environments + neutral one + cognitive task	Final debriefing

Table 2. Study protocol for the control group.

Intake session	Introduction of the training	Clinical interview (MINI)	Give STAI Y2, PMS, PSS, COPE and SWLS	Questionnaire about participants'	Explanation of how to use the diary

					expectations and motivations		
Session I	Physiological assessment	Give STAI Y1 and VAS-A (baseline)	Guided imagery to stressful environment n1	Give STAI Y1 and VAS-A (after each scenario)	Repeated for all the 7 stressful environments + neutral one + cognitive task	Exposition to a relaxing virtual environment	Final debriefing
Assessment session							
Session II	Preliminary phase	Pre-training	Training			Post-training	Final phase
Training session	Give STAI Y1 and VAS-A	Physiological assessment	Guided imagery to stressful environment	Cognitive restructuring	Guided imagery to a relaxing environment	Give STAI Y1 and VAS-A	Delivery of homework and debriefing
Session III	Preliminary phase	Pre-training	Training			Post-training	Final phase
Training session	Give STAI Y1 and VAS-A	Physiological assessment	Guided imagery to stressful environment	Cognitive restructuring	Guided imagery to a relaxing environment	Give STAI Y1 and VAS-A	Delivery of homework and debriefing
Session IV	Preliminary phase	Pre-training	Training			Post-training	Final phase
Training session	Give STAI Y1 and VAS-A	Physiological assessment	Guided imagery to stressful environment	Cognitive restructuring	Guided imagery to a relaxing environment	Give STAI Y1 and VAS-A	Delivery of homework and debriefing
Session V	Preliminary phase	Pre-training	Training			Post-training	Final phase
Training session	Give STAI Y1 and VAS-A	Physiological assessment	Guided imagery to stressful environment	Cognitive restructuring	Guided imagery to a relaxing environment	Give STAI Y1 and VAS-A	Delivery of homework and debriefing

Session VI	Preliminary phase		Pre-training	Training			Post-training	Final phase
Training session	Give STAI Y1 and VAS-A		Physiological assessment	Guided imagery to stressful environment	Cognitive restructuring	Guided imagery to a relaxing environment	Give STAI Y1 and VAS-A	Delivery of homework and debriefing
Session VII	Preliminary phase		Pre-training	Training			Post-training	Final phase
Training session	Give STAI Y1 and VAS-A		Physiological assessment	Guided imagery to stressful environment	Cognitive restructuring	Guided imagery to a relaxing environment	Give STAI Y1 and VAS-A	Delivery of homework and debriefing
Session VIII	Preliminary phase		Pre-training	Training			Post-training	Final phase
Training session	Give STAI Y1 and VAS-A		Physiological assessment	Guided imagery to stressful environment	Cognitive restructuring	Guided imagery to a relaxing environment	Give STAI Y1 and VAS-A	Delivery of homework and debriefing
Session IX	Preliminary phase		Pre-training	Training			Post-training	Final phase
Training session	Give STAI Y1 and VAS-A		Physiological assessment	Guided imagery to stressful environment	Cognitive restructuring	Guided imagery to a relaxing environment	Give STAI Y1 and VAS-A	Delivery of homework and debriefing
Session X<br>Final session	Follow up of questionnaires used in the evaluation phase	Physiological assessment	Give STAI Y1 and VAS-A	Guided imagery to stressful environment<br>n 1	Give STAI Y1 and VAS-A	Repeated for all the 7 stressful environments + neutral one + cognitive task	Exposition to a relaxing virtual environment	Final debriefing
